# Characterization of the Myocardial Inflammatory Response in Acute Stress-Induced (Takotsubo) Cardiomyopathy

**DOI:** 10.1016/j.jacbts.2018.08.006

**Published:** 2018-12-31

**Authors:** Heather M. Wilson, Lesley Cheyne, Paul A.J. Brown, Keith Kerr, Andrew Hannah, Janaki Srinivasan, Natallia Duniak, Graham Horgan, Dana K. Dawson

**Affiliations:** a School of Medicine, Medical Sciences & Nutrition, University of Aberdeen, Foresterhill, Aberdeen, Scotland, United Kingdom; b Department of Pathology, Aberdeen Royal Infirmary, Foresterhill, Aberdeen, Scotland, United Kingdom; c Department of Cardiology NHS Grampian, Aberdeen Royal Infirmary, Foresterhill, Aberdeen, Scotland, United Kingdom

**Keywords:** histopathology, inflammation, macrophage, pathophysiology, takotsubo cardiomyopathy, EF, ejection fraction, IL, interleukin, MHC, major histocompatibility complex, MI, myocardial infarction, qPCR, quantitative polymerase chain reaction, TNFα, tumor necrosis factor-alpha

## Abstract

Takotsubo cardiomyopathy is an acute stress-induced heart failure syndrome for which the exact pathogenic mechanisms are unclear, and consequently, no specific treatment exists. In an experimental model of stress-induced takotsubo-like cardiomyopathy, the authors describe the temporal course of a chronic inflammatory response post-induction, with an initial early influx of neutrophils into myocardial tissue followed by macrophages that are typical of a proinflammatory M1 phenotype, and a nonsignificant increase in systemic inflammatory cytokines. Post-mortem myocardium from the more complex clinical takotsubo patients share features of the study’s experimental model. These findings suggest modulators of inflammation could be a potential therapeutic option.

## Highlights

• Takotsubo cardiomyopathy is an acute heart failure syndrome often triggered by emotional or physical stress, where no treatment currently exists, and exact pathogenic mechanisms are unclear.
• Rats in which takotsubo-like cardiomyopathy was induced showed localized myocardial inflammatory changes, including progressive inflammatory infiltrates and myofiber atrophy, that persisted over the 14-day time course examined.
• Early neutrophil infiltrates were followed by clusters of myocardial macrophages, typically of an M1 proinflammatory phenotype, with no switch to M2 resolving macrophages; individual M2 macrophage levels, however, correlated with recovery in cardiac function. Human post-mortem myocardial tissue shared features of the experimental model demonstrating M1 macrophage clusters.
• The persistent clinical symptoms and long-term morbidity/mortality observed in takotsubo patients may, in part, relate to chronic nonresolving myocardial inflammation.

## Visual Abstract

**Figure:**
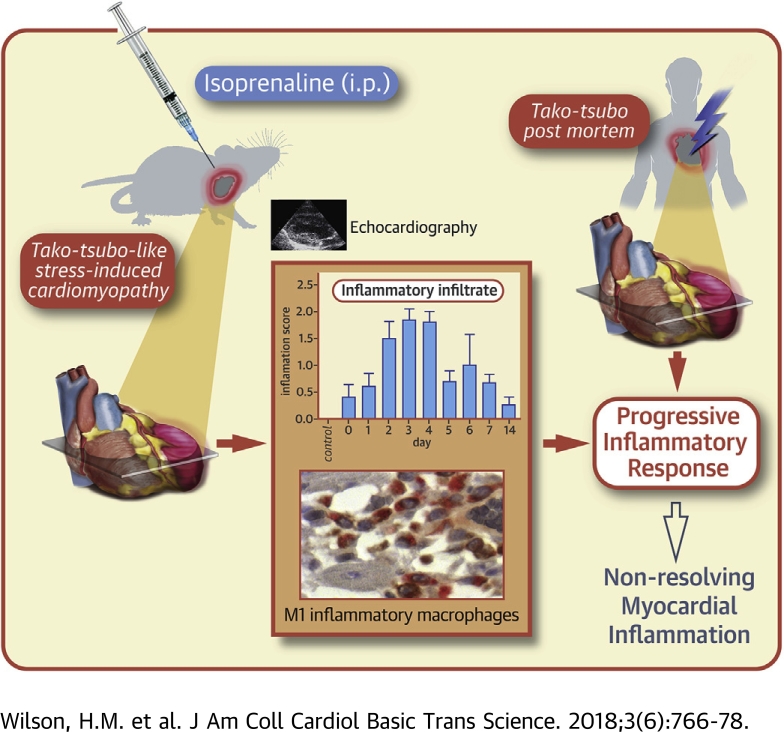

Acute stress-induced (takotsubo) cardiomyopathy has a dramatic clinical presentation, mimicking a myocardial infarction (MI) (1) and is often precipitated by major emotional/physical stress. Despite unobstructed coronary arteries, the left ventriculogram shows characteristic myocardial ballooning, not corresponding to a single coronary artery territory, and the heart function is severely reduced. Regardless of a spontaneous process of recovery as a recognized feature of takotsubo cardiomyopathy, the mortality risk and long-term mortality are also comparable with MI 2, 3. There is no current treatment, and the etiology is unclear, therefore its pathophysiology needs to be resolved before targeted therapies can be trialed. We have previously demonstrated severe global edema in both left and right ventricular myocardium of takotsubo patients during the acute phase with incomplete resolution at 4 months 4, 5, 6, 7. The substrate of this persistent edema is most likely an ongoing inflammatory response, which remains to be characterized in detail. However, this is challenging in patients, where coexistent cardiac pathologies, lack of access to tissue, very small biopsies that do not reflect global changes, and tissue availability only at a single time point, are the norm.

Here, we used a previously established and well-reported rodent experimental model of catecholamine (stress)-induced takotsubo-like cardiac dysfunction 8, 9, 10, 11, 12, where the disease process is consistent and reproducible, and there are no confounding complications or major limitations to tissue availability. Although it remains unclear whether a “catecholamine surge” is fundamentally causal in triggering human takotsubo cardiomyopathy, this experimental model results in a similar wall motion abnormality that is observed in the human disease in the absence of an ischemic or infectious insult 8, 9, 10, 11, 12. Our aim was to establish the type and time course of the myocardial inflammatory changes in this stress-induced experimental model and to then determine whether similar changes existed in viable, nonfibrotic myocardium from necessarily more complex human post-mortem cases. We specifically focused on detecting and characterizing macrophages, a heterogeneous cell population that infiltrates damaged tissue and functionally range between proinflammatory (M1 macrophages) and anti-inflammatory/tissue reparative/profibrotic (M2 macrophages) subtypes 13, 14. Our results reveal, for the first time to our knowledge, a clear, localized, and temporal inflammatory response with a predominance of M1 proinflammatory macrophages within rat myocardial tissue following induction of takotsubo-like cardiomyopathy and evidence for a similar process in more complex human post-mortem cases. Unlike post-MI, there is no switch to M2-resolving macrophages, and the percentage of M2 macrophages correlates with recovery of cardiac function. This low-level, nonresolving inflammation provides a rationale for an immunomechanistically targeted therapeutic strategy in a condition for which no current therapy is available.

## Methods

### Animals

All animal work was performed in accordance with the United Kingdom Home Office regulations and approved by Aberdeen University Animal Ethics Committee. Female Sprague Dawley rats, 4 to 6 months old (due to better survival rates on disease induction than in older rats), weighing 300 ± 12 g were housed in a temperature-controlled (25°C) facility with a 12-h light/dark cycle and given free access to food and water.

### Human post-mortem samples

Research approval for post-mortem hearts was obtained under family consent for 2 female patients (69 and 54 years of age) who died shortly after episodes of acute takotsubo cardiomyopathy in Aberdeen Royal Infirmary. Patient 1 presented with chest pain, and an electrocardiogram showed 9-mm ST-segment elevation in inferior leads and ST-segment depression in the corresponding precordial leads; cardiac catheterization showed normal coronary arteries and a large area of apical and mid-cavity ballooning (Supplemental Figure 1A). She died 5 h after presentation. Autopsy established apical inferior myocardial rupture with tamponade and 50% stenosis of the right coronary ostium. Patient 2 had a previous, documented takotsubo event at a family funeral 3 years before death, and presented at another family funeral with chest pain. There was anterior ST-segment elevation on electrocardiogram, moderate stenosis in the right coronary artery on catheterization, and apical ballooning on left ventriculogram (Supplemental Figure 1B). She died on the fifth day of admission, in torsade de pointes and ventricular fibrillation. Autopsy established patent coronary arteries with moderate atheroma of the right coronary artery and an old area of left ventricular myocardial fibrosis. Both patients had a modest, disproportionately low biomarker (troponin) rise relative to the large area of wall motion abnormality. No recent infarction was noted on microscopy at autopsy. The myocardial tissue used for analysis was from areas unaffected by fibrosis. Four control heart specimens with no known clinical or post-mortem findings of heart disease were obtained from archived cases. Formalin-fixed myocardium (5-μm sections) were examined by histology and immunohistochemistry and scored in a blinded fashion by P.A.J.B. and N.D.

### Induction of stress-induced cardiomyopathy

Rats received a single dose of 100 mg/kg isoprenaline intraperitoneally, a protocol that induces reproducible takotsubo-like cardiac dysfunction 8, 9, 10, 11, 12 with widespread akinesia or hypokinesia of apical segments and hypercontractility of the cardiac base in 85% of rats. This model results in supraphysiological levels of circulating catecholamines, initial high blood pressure and heart rates, and a high yield of wall motion abnormality after the induced stress of isoprenaline and thus provides a stress-induced model in the absence of ischemic and pathogen-induced inflammatory insults. Systolic regional wall motion abnormalities in this model disappear between days 6 to 15 post-induction. Survival rates following injection were 90%. Similar volumes of intraperitoneal saline injections were given to the control rat group. Rats were sacrificed serially on day 0 (6 h after injection) and days 1 to 7 and day 14 after disease induction, with group sizes as indicated for each experimental analysis.

### Echocardiography

Echocardiography was performed before sacrificing rats to ascertain the presence of wall motion abnormality and to quantify the cardiac function. A Vivid Q equipment (GE Healthcare, Oslo, Norway) with an 11.5-MHz pediatric probe was used to acquire images. Rats were placed under anesthesia (1.25% isoflurane in oxygen after 4% isoflurane induction), and chests were shaved. One long-axis and 3 short-axis views (base, mid, and apex) were acquired. The duration of anesthesia did not exceed 2 min in any rat. Heart rate was 290 ± 5 beats/min during echocardiography (normal rate). Ejection fraction (EF) was calculated using the area–length method (15). Characteristics of experimental stress-induced cardiomyopathy are shown in Supplemental Figure 2.

### Rat heart tissue processing

After schedule 1 sacrifice, rat hearts were fixed in 5% phosphate-buffered formaldehyde and embedded in paraffin. All hearts were sectioned in 3 transverse blocks: apex, mid-heart, and base, and 4-μm sections from each level examined by histology and immunohistochemistry. Isoprenaline-injected rats for each of the time points (day 0 equivalent to 6 h after injection), days 1 to 7 and day 14 (n = 7 per group) was compared with saline-injected control rats (n = 7 per group), that is, 70 animals in total. Because some deaths occurred on injection in our designed groups, the number of animals available for each group was 5 to 7 animals as indicated in *Results*. In additional experiments, hearts were snap frozen for gene expression analysis (control, saline-injected animals, and animals sacrificed on day 3 and day 14 after induction of takotsubo cardiomyopathy).

### Histopathology

Hematoxylin and eosin–stained sections from the mid-heart, where the wall motion abnormality and subendocardial hemorrhage were observed, were scored semiquantitatively in a blinded manner for histological/pathological changes by an experienced consultant pathologist (K.K.). Arbitrary scores of 0, 1, and 2 were given for hemorrhage, immune/inflammatory cellular infiltrates, eosinophilic fiber staining areas, and degenerate myofibrils/fiber atrophy where the scoring system was defined as 0 = no lesions, 1 = scattered/patchy/little present, and 2 = readily identifiable throughout the section. In some sections, hemosiderin was identified in macrophages and confirmed by Prussian blue stain, and was scored on a scale of 0 to 2 (Supplemental Figure 3). Most insults occurred in regional areas of the section as illustrated in Supplemental Figure 3.

### Inflammatory cell composition: Immunohistochemistry

Sections were deparaffinized, microwaved in citrate buffer (pH 6.0), and endogenous peroxidase activity quenched by incubation with 3% H_2_O_2_. Neutrophil counts were determined from hematoxylin and eosin–stained sections by morphology. Macrophages and T cells were detected by anti-CD68 antibodies (ED1, rat and KP1, human) (Abcam, Cambridge, United Kingdom) and anti-CD3 antibodies (MCA772GA, rat, Bio-Rad, Hercules, California; and F7.2.38, human, Dako, Glostrup, Denmark), respectively, and B cells by anti-CD20 antibody, L26 (Abcam) and the DAKO Envision Detection Kit K5007. Specific brown staining was developed by incubation with Chemate-DAB (Dako, Ely, United Kingdom). Slides were counterstained with hematoxylin, and nuclei blue stained with Scott’s Tap Water. The DAKO G2-Double staining kit, K5361 (DakoCytomation, Glostrup, Denmark) was used for staining with anti-CD68 (ED1, rat and KP1, human) and the M1-macrophage markers anti-major histocompatibility complex (MHC) class II (OX-3, rat, TAI.1B5, human) and inducible nitric oxide synthase (iNOS) (AB15323; Bio-Rad) for rat, and SOCS3 (AB16030, Abcam) for human and the M2-macrophage marker, anti-CD163 (ED2, Abcam for rat; EDHu-1, Bio-Rad for human), according to the manufacturers’ instructions. Positive staining was detected using diaminobenzidine/Liquid Permanent-Red (DakoCytomation) (16). A negative control where primary antibody was replaced by a nonspecific IgG equivalent was included in all analyses.

### Quantification of neutrophil, macrophage, and T-cell counts

The mid-heart sections of rat hearts was partitioned into 8 consecutive sections (Supplemental Figure 4), and these were used to quantify macrophage, T-cell, and neutrophil counts. Each section was separated into 3 fields of view (epicardium, mid-wall, and endocardium) to directly compare areas. For human post-mortem tissue, defined sections approximately 3 cm × 2 cm of myocardial tissue was used. Intact CD68-positive cells were counted, and absolute counts represent the mean number of macrophages from each of the 8 sections of rat tissue. The entire area of human post-mortem tissue was considered, and the average macrophage number per field determined from 15 fields per section was determined. Counts were repeated until 3 consecutive values were obtained with a margin of error of ±5%. A similar technique was used to count the CD3-positive T cells and neutrophils per area. Both the intraobserver and interobserver variation were found to be <5% (p < 0.002). The number of double-positive cells were counted and expressed as a percentage of total macrophages.

### Quantification of serum and tissue cytokines

Serum samples collected from blood derived on cardiac puncture at the time of sacrifice were analyzed for tumor necrosis factor (TNF)-α, interleukin (IL)-6, and IL-10 as determined by DuoSet enzyme-linked immunosorbent assay kits from R&D Systems (Minneapolis, Minnesota) according to the manufacturer’s instructions. Cytokine levels in the myocardial tissue were determined by quantitative polymerase chain reaction (qPCR). Tissues were homogenized in TRIzol reagent (Sigma-Aldrich, St. Louis, Missouri) and cDNA synthesis carried out from 1 mg of RNA using a Tetro cDNA Synthesis Kit (Bioline, London, United Kingdom). qPCR was performed using a Light Cycler 480 (Roche, Basel, Switzerland), and gene expression of TNF-α, il-6 and il-10, was determined in relation to housekeeping gene *HPRT*. Groups analyzed consisted of control, saline-injected animals (n = 10), isoprenaline-injected animals sacrificed 3 days after injection (n = 9), and isoprenaline-injected animals sacrificed 14 days after injection (n = 5).

### Statistical analyses

The Mann-Whitney *U* test was used to compare the macrophage, neutrophil, and T-cell counts/heart section. Pearson’s correlation coefficient determined the significance of a correlation between macrophages and their subtypes with heart EF. All values are reported as mean ± SD. A value of p < 0.05 was considered as significant.

## Results

### Histological characterization of inflammation in stress-induced takotsubo-like cardiomyopathy

**Figure 1:**
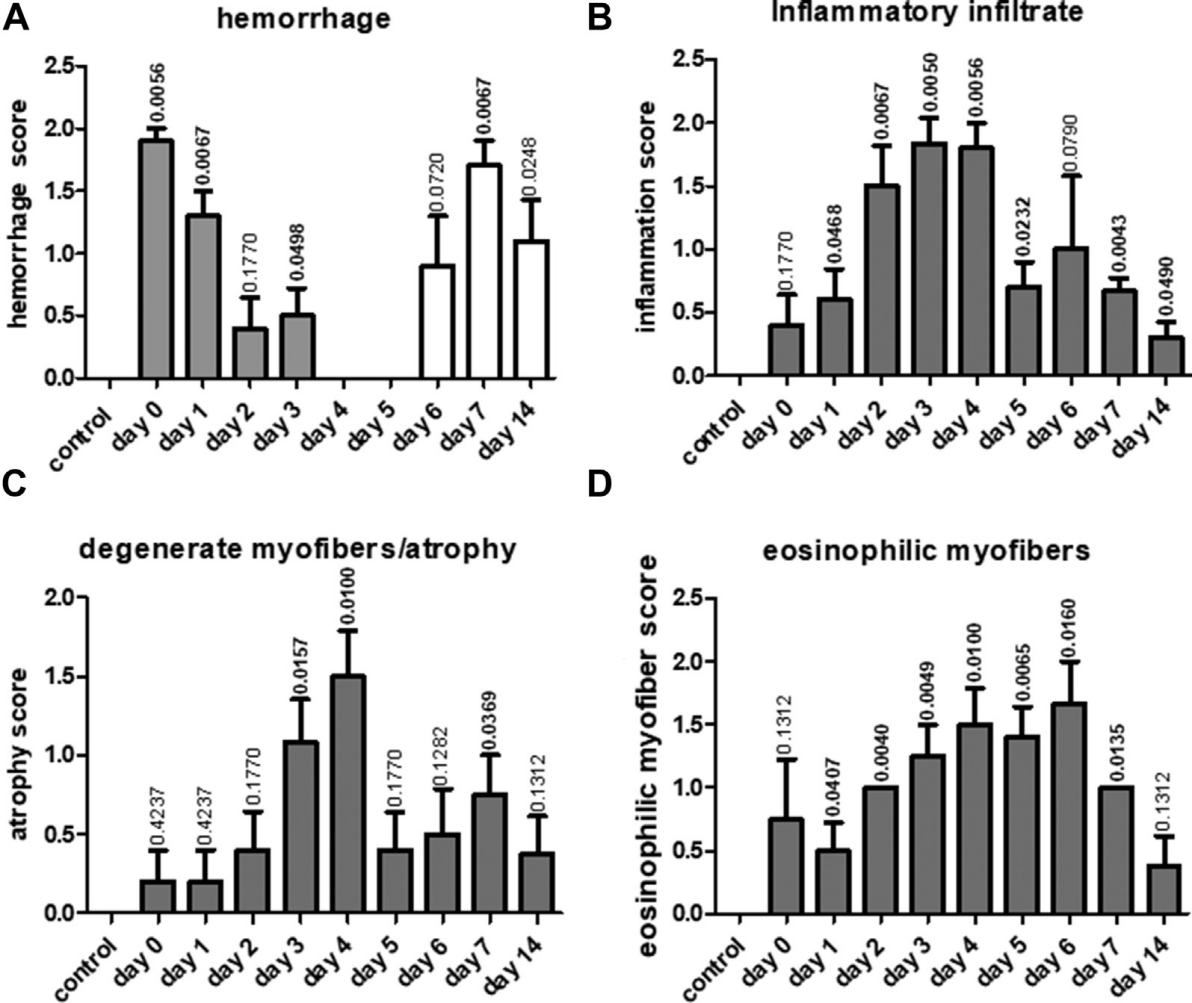
Histological Characterization of Mid-Heart Sections of Control Rats and From Rats After Induction of Takotsubo Cardiomyopathy Histological characterization of hematoxylin and eosin–stained mid-heart sections of control rats and from rats over the time course (0 to 14 days) after induction of takotsubo cardiomyopathy show a temporal immune/inflammatory cellular infiltrate. Sections of heart tissue were semiquantitatively scored by a consultant histopathologist (K.K.) blinded to the study. Arbitrary scores of 0, 1, and 2 were given for **(A)** hemorrhage **(gray bars)** or presence of hemosiderin-containing phagocytic cells **(white bars)**, **(B)** immune/inflammatory cellular infiltrates, **(C)** degenerate myofibrils/myofiber atrophy, and **(D)** eosinophilic myofibers. The number of animals per group were as follows; control n = 7, day 1 n = 6, day 2 n = 6, day 3 n = 7, day 4 n = 6, day 5 n = 7, day 6 n = 6, day 7 n = 5, and day 14 n = 5 animals. Statistical difference in histological scores between control rats and those injected with isoprenaline were calculated with p values denoted above each time point bar. Values in bold indicate statistically significant differences compared with the control group.

To determine initially the type and time course of the myocardial inflammatory changes in the experimental model of stress-induced cardiomyopathy, sections of rat heart were characterized histologically. Six hours after administration of isoprenaline to rats, histopathological examination revealed localized subendocardial hemorrhage in the mid-ventricular region of the heart (maximum histopathological score 1.9) (Figure 1A) and post-day 5 showed an increase in hemosiderin-containing phagocytic cells (confirmed by Prussian blue stain) (Supplemental Figure 3D), supporting a resolution of injury. An immune cell/inflammatory infiltrate was evident after 6 h, peaking at days 3 to 4 showing scattered, but later, obvious infiltrates in regional areas, subsiding by day 5 (Figure 1B, Supplemental Figure 3). This infiltrate correlated with prevalence of degenerate myofibers where histological scores peaked at day 4, subsiding at day 5. Beyond day 5, fiber atrophy was more evident, then decreased by day 14 (Figure 1C). Hypereosinophilic myofibers were observed, peaking at day 6 when nuclear pyknosis was observed (Figure 1D), although this change was found across the 14-day period examined. Fibrosis was also identified in late-stage sections (days 6, 7, and 14), consistent with the sequelae of earlier inflammatory changes. Vascular proliferation was found in 50% of animals by day 14. No gross changes in monocyte numbers or histology of the spleen could be detected between the animal groups.

### Detailed characterization of immune cell infiltrate over time

**Figure 2:**
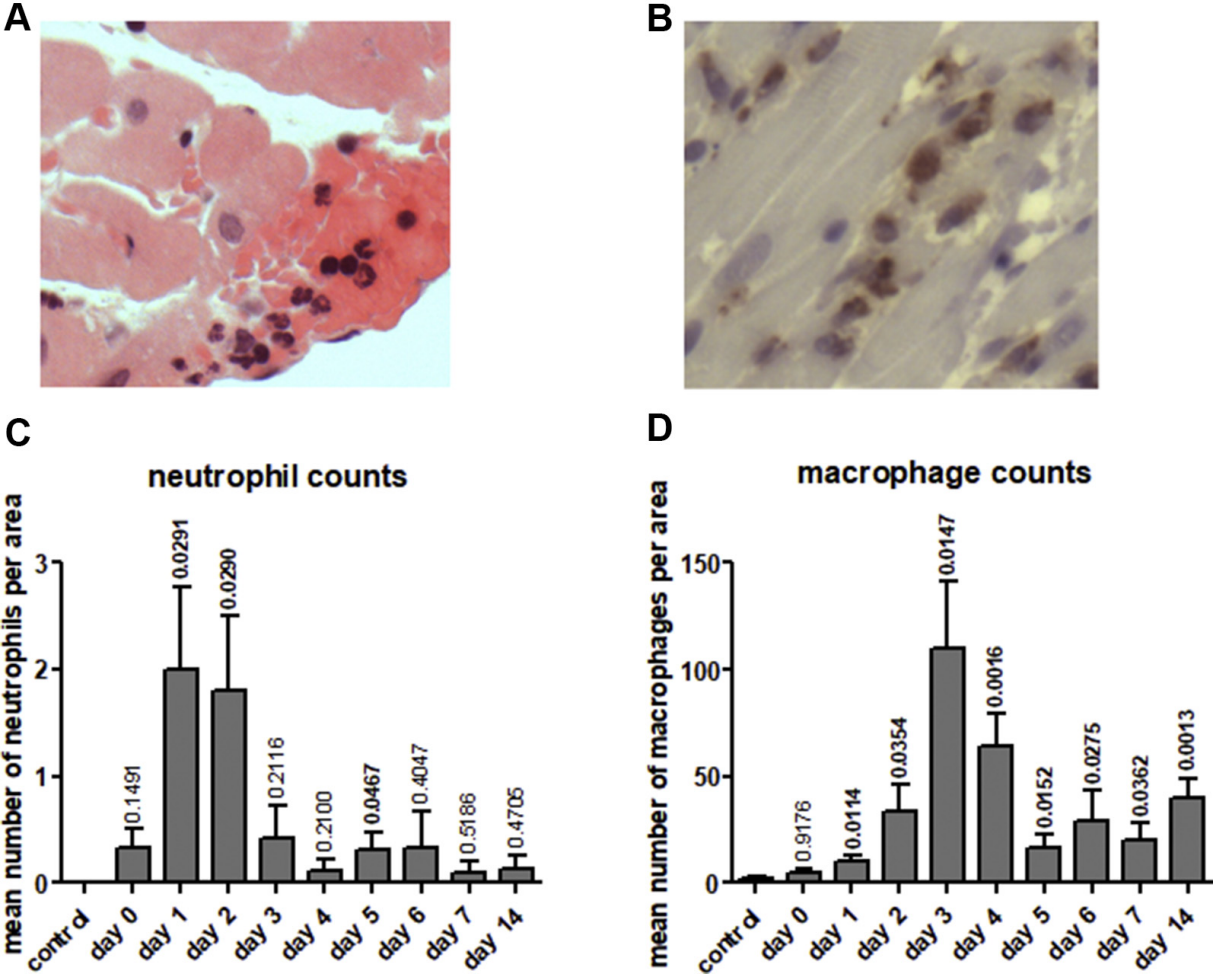
Immunohistochemical Analysis Following Induction of Stress-Induced Takotsubo-Like Cardiomyopathy Immunohistochemical analysis demonstrates progressive neutrophil and macrophage infiltration into myocardial tissue (day 0 to 14) following induction of stress-induced takotsubo-like cardiomyopathy. Representative examples of neutrophil **(A)** and macrophage (CD68-positive cells) **(B)** staining in mid-region rat myocardial tissue, original magnification ×400. Neutrophil **(C)** and macrophage **(D)** cell counts in the mid-region of saline injected (control) and isoprenaline injected rat hearts (day 0 to 14). Values represent mean counts ± SD in 24 fields of view from defined areas as described in the Methods section. The number of animals per group were as follows: control n = 7, day 1 n = 6, day 2 n = 6, day 3 n = 7, day 4 n = 6, day 5 n = 7, day 6 n = 6, day 7 n = 5, and day 14 n = 5 animals. Statistical difference in mean number of neutrophil and macrophage counts between control rats and those injected with isoprenaline were calculated with p values denoted above each time point bar. Values in bold indicate statistically significant differences compared with the control group.

The increase in immune/inflammatory cell infiltrates observed by histological analysis over the time course of disease was examined in more detail by specific cell characterization defining the macrophages, neutrophils, and T-cell counts within the rat mid-heart sections. Six hours following isoprenaline injection, early neutrophilic infiltrates predominated, peaking at day 1 to 2 and subsiding thereafter, typical of an inflammatory response (Figure 2). Macrophage counts peaked at day 3 and were typically found in regional clusters. Macrophage infiltrates subsided by day 5, but were still evident at day 14 and did not return to baseline levels, consistent with the time scale of the immune cell infiltrate noted on histological assessment. We observed no detectable T-cell infiltrates over the time course examined.

Cardiac function as determined by the change in percentage EF pre- and post-isoprenaline injection, deteriorated immediately after disease induction, and partially recovered thereafter, although not to baseline (Supplemental Figure 2). The most significant decline in cardiac function was observed at the 6-h post-induction time point and thus preceded macrophage infiltration and the time of peak inflammatory cell infiltrate.

### Comparisons of the proportion of M1 and M2 macrophage subtypes over the course of disease

**Figure 3:**
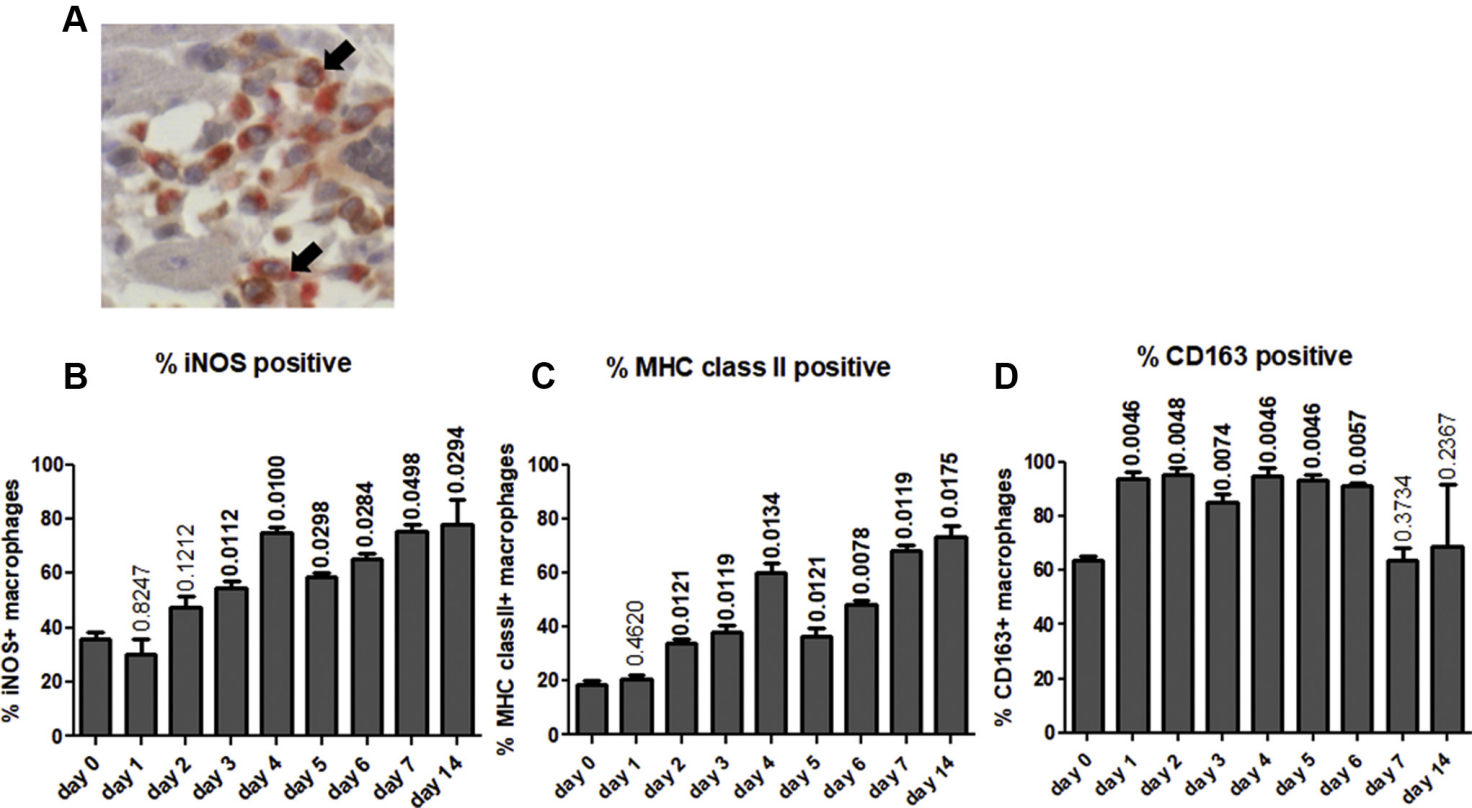
Double Immunostaining of Mid-Heart Sections Showing M1-Activated and M2-Positive Macrophages Double immunostaining of mid-heart sections show a greater predominance of M1-activated macrophages over time with little change in percentage of M2-positive macrophages. Total macrophages were detected by anti-CD68 and liquid permanent red staining **(red)**, whereas macrophage activation markers were identified by the relevant antibodies and DAB staining **(brown)**. Macrophage-rich areas were analyzed as outlined in *Methods*. Examples of double-stained macrophages are highlighted by **arrows in A**. Original magnification ×400. Changes in percentages of double-stained macrophages over time for inducible nitric oxide synthase (iNOS) **(B)**, major histocompatibility complex (MHC) class II **(C)**, and CD163 **(D)**. The number of animals per group were as follows; control n = 6, day 1 to 14 n = 5 for each time point. Statistical difference in the percentage iNOS, MHC class II, and CD163-positive macrophages between control rats and those injected with isoprenaline were calculated with p values denoted above each bar. Values in bold indicate statistically significant differences compared with the control group.

Given the increase in macrophage numbers in heart sections of our experimental model of takotsubo-like cardiomyopathy, it was important to characterize their inflammatory phenotype. The proportions of M1 (proinflammatory tissue destructive macrophages expressing markers iNOS and MHC class II) and M2 (CD163-positive anti-inflammatory, tissue reparative/profibrotic macrophages) 11, 12 were therefore examined (Figure 3A).

The percentage of MHC class II–positive macrophages and iNOS-positive macrophages, indicative of M1 cells, increased over time after disease induction (Figures 3B and 3C), peaking at day 4. The percentages decreased between day 4 and 5 and then demonstrated a second peak of increase at days 6 to 14 consistent with the increase in total macrophage counts at that time and a classic inflammatory response.

The percentage of CD163-positive M2 macrophages showed a very different pattern. They increased from day 0 to day 1, suggesting increased macrophage activation. The percentage did not change significantly from day 1 to day 6, but fell on day 7 of disease (Figure 3D).

**Figure 4:**
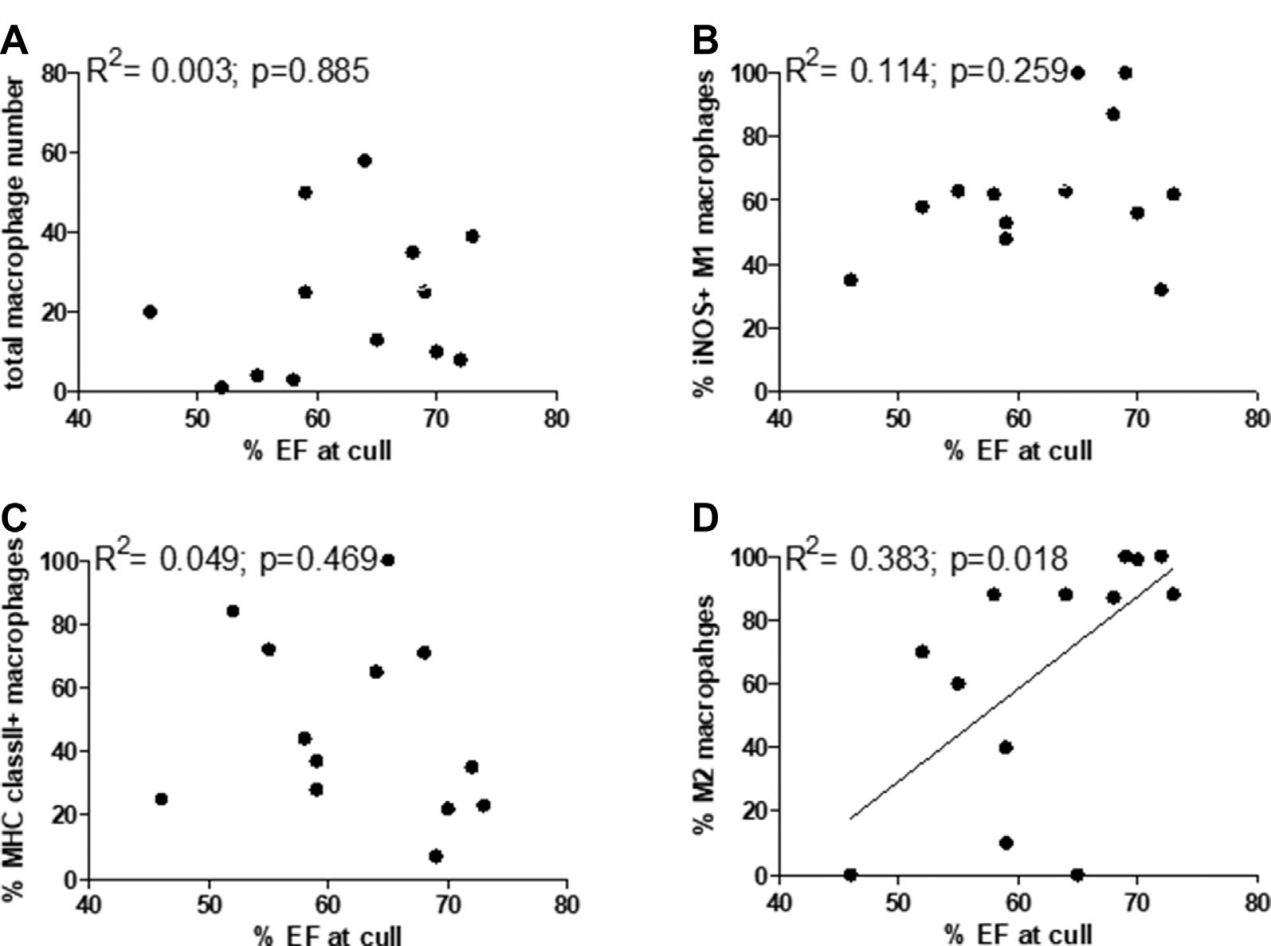
Correlations Between the Total Macrophage Counts or the Percentage M1 and M2 Macrophages and Recovery of Cardiac Function Correlations between the total macrophage counts or the percentage M1 and M2 macrophages and recovery of cardiac function as determined by percentage left ventricular ejection fraction (EF). The total number of macrophages **(A)** or percentage of iNOS **(B)** and MHC class II–positive **(C)** M1 macrophages and CD163-positive **(D)** M2-like macrophages was determined from immunohistochemical analysis and correlated with the percentage ejection fraction before cull. A total of 13 animals were analyzed. The coefficient of determination (R^2^) and p value were determined by Pearson’s correlation test.

To determine whether there was a relationship between the total macrophage counts or the percentage of M1 and M2 macrophages and recovery of cardiac function, we correlated the macrophage numbers or M1/M2 percentages with percentage left ventricular EF determined before culling (Figure 4). There was no significant correlation between the level of total myocardial macrophage infiltration or percentage M1 macrophages and the heart EF (R^2^ = 0.003; p = 0.885 total macrophages; R^2^ = 0.114; p = 0.259, iNOS-positive M1 macrophages; and R^2^ = 0.049; p = 0.469, MHC class II–positive M1 macrophages). There is a significant correlation (R^2^ = 0.383; p = 0.018) between the percentage of M2 macrophages and percentage EF, indicating that a greater preponderance of M2 cells improved recovery of cardiac function.

### Local and systemic cytokine changes over the course of disease

**Figure 5:**
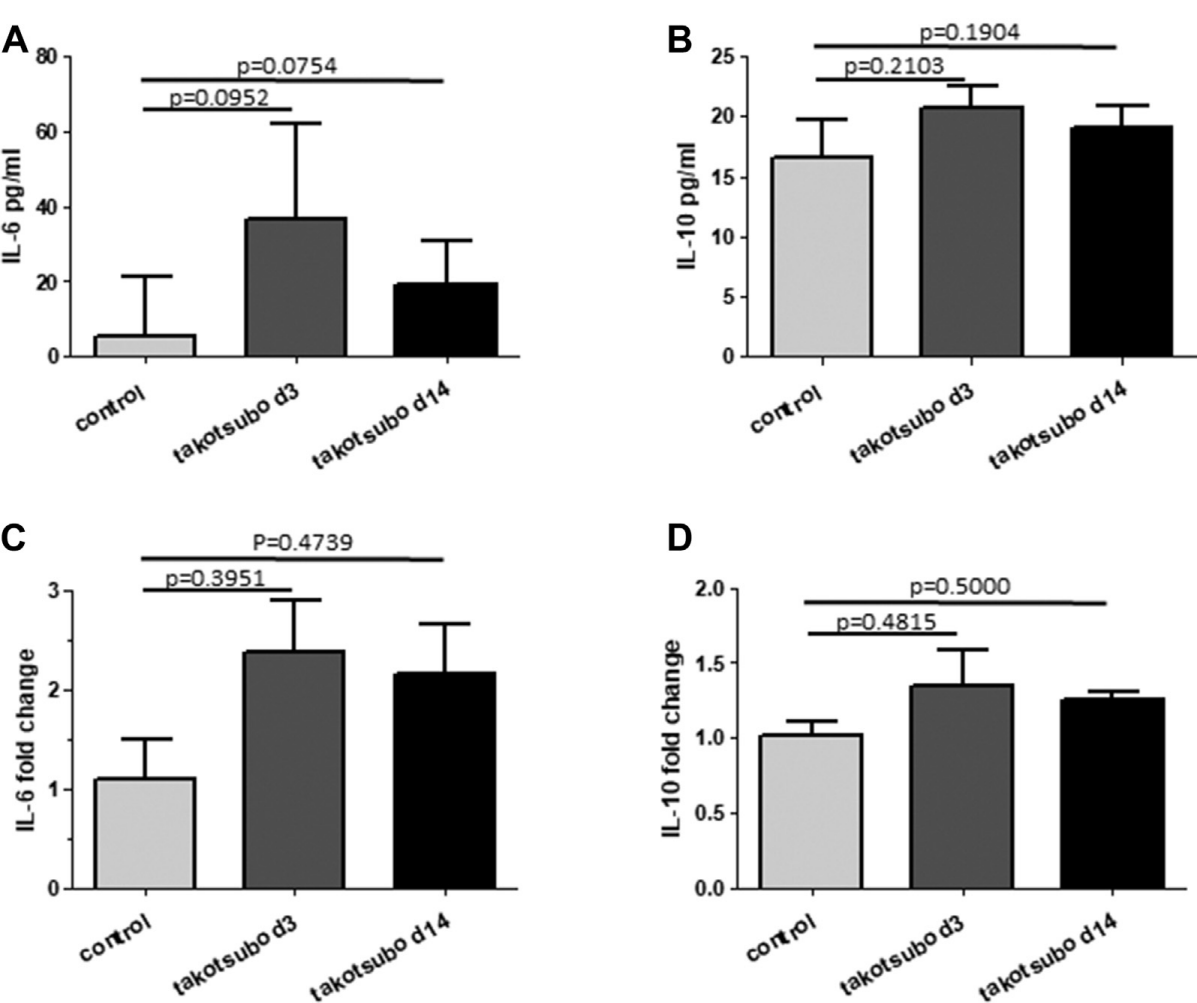
Analysis of Systemic and Myocardial Cytokine Levels in Control Animals and Those With Takotsubo-Like Cardiomyopathy Analysis of systemic and myocardial cytokine levels in control animals and those with Takotsubo-like cardiomyopathy at day 3 (d3) and day 14 (d14) are shown. Serum levels of IL-6 **(A)** and IL-10 **(B)** are mean pg/ml levels ± SD; gene expression levels of IL-6 **(C)** and IL-10 **(D)**. Groups analyzed consisted of control, saline-injected animals (n = 10), isoprenaline-injected animals sacrificed 3 days after injection (n = 9), and isoprenaline-injected animals sacrificed 14 days after injection (n = 5). Values are mean fold change over control animals ± SD. Values above each bar indicate p values compared with control animals. IL = interleukin.

The levels of pro- and anti-inflammatory cytokines were also analyzed post-isoprenaline injection and compared with control rats to determine whether local and systemic inflammation was apparent and could contribute to the underlying macrophage polarization. Systemic cytokine levels were low (IL-6 and IL-10) or undetectable (TNF-α) with or without induction of takotsubo-like cardiomyopathy. At day 3 and day 14 post-induction, IL-6 and IL-10 systemic levels were elevated over control animals (Figures 5A and 5B), although this did not reach statistical significance (IL-6 day 3 p = 0.0952, IL-6 day 14 p = 0.0754; IL-10 day 3 p = 0.2103, IL-10 day 14 p = 0.1904). Gene expression levels for IL-6 and IL-10 in myocardial tissue were also greater post-disease induction (Figures 5C and 5D), but again, this increase did not reach statistical significance (IL-6 day 3 p = 0.3951, IL-6 day 14, p = 0.4739; IL-10 day 3 p = 0.4815, IL-10 day 14 p = 0.5000). Gene expression levels for TNFα were on the limit of detection by qPCR for both control and takotsubo-like cardiomyopathy animals (data not included).

Overall, these results in our experimental model suggest a characteristic ongoing inflammatory response within the rat myocardium, with early and prominent neutrophil infiltration, followed by M1 proinflammatory macrophages.

### Characterization of inflammatory responses in human post-mortem takotsubo cardiomyopathy patients

To determine whether such an inflammatory component observed in a rat model could also be identified in post-mortem samples of human takotsubo cardiomyopathy patients, myocardial tissue from 2 patients (Patient #1 deceased 5 h and Patient #2 deceased 5 days post-presentation) was assessed. The myocardium used for assessment was unaffected by any visible macroscopic fibrotic changes or infarction. Occasional small areas of lymphocytic and macrophage infiltration were evident in the interstitium and within areas of myocytes underlying the pericardial fat. Focal subendocardial hemorrhage was also present. For control hearts with no known heart condition and no post-mortem findings of heart disease, less prominent lymphocytic and macrophage infiltration was observed histologically in all cases (n = 4).

**Figure 6:**
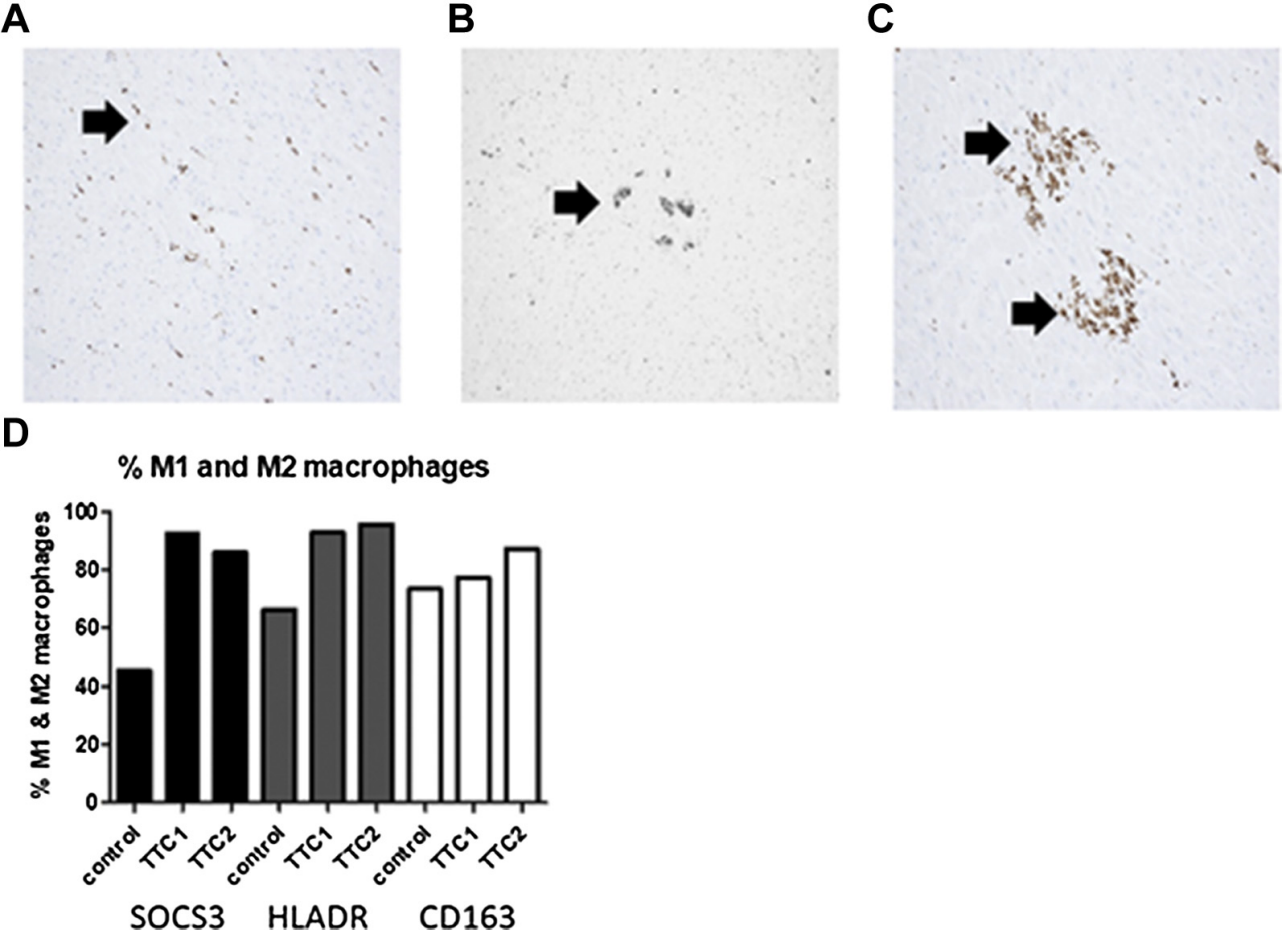
Post-Mortem Myocardial Tissue From Takotsubo Patients Post-mortem myocardial tissue from takotsubo patients show the presence of macrophages and demonstrate an increase/predominance of M1 macrophages. Macrophage staining (CD68-positive cells) showing, as indicated by **arrows**: **(A)** scattered macrophages in the subendothelial area of the myocardium in a control subject with no underlying heart condition, **(B)** very few subendothelial aggregates of macrophages in patient 1 deceased 5 h after presentation with takotsubo cardiomyopathy, and **(C)** subpericardial aggregates of macrophages in the myocardial tissue from patient 2 with takotsubo cardiomyopathy deceased 5 days after presentation. Original magnification ×400. **(D)** Percentage of CD68-positive macrophages staining for M1 markers HLADR and SOCS3, and M2 marker CD163 in patients with takotsubo cardiomyopathy (TTC) (n = 2) and age/sex-matched control subjects (control) (n = 4).

Immunohistochemical analysis demonstrated a scattered distribution of CD3-positive cells in both takotsubo cardiomyopathy patients’ post-mortem myocardial tissues, whereas control tissues demonstrated sparse cell positivity and 1 case that revealed scattered groups of CD3-positive cells. Control tissue showed no positive staining for CD20 (B lymphocytes), whereas both patients revealed a scattered CD20 cell positivity. Patient #2, deceased 5 days following presentation, demonstrated a scattered macrophage distribution with occasional aggregated clusters of macrophages in the subendothelium and subpericardial regions (Figure 6), whereas aggregated clusters were less obvious in Patient #1, deceased 5 h after onset of symptoms or in control subject myocardium.

More quantitative analysis of macrophage counts showed the average macrophage number per field in patients was significantly higher than in control subjects (10.6 ± 2.8 Patient #1; 27.01 ± 16.1 Patient #2, vs. 6.7 ± 5.4 control tissue; p < 0.05). To compare the M1/M2 phenotypes of macrophages between groups and relate results to that of our experimental model, double immunohistochemistry was performed. As with our experimental model, there was a greater proportion of M1 macrophages (CD68/HLADR or CD68/SOCS3 positive) in myocardial tissue from takotsubo cardiomyopathy patients when compared with control tissue, again reflecting a potential proinflammatory response (Figure 6). By contrast, there was no obvious difference in the proportion of M2, CD163-positive myocardial tissue macrophages between patients and control subjects.

## Discussion

Several mechanisms have been implicated in pathogenesis of takotsubo cardiomyopathy, including coronary artery spasm, microvascular dysfunction, and excessive release of catecholamines in response to stress 17, 18. None of these mechanisms, however, has been conclusively proven to be responsible for the disease solely or in combination, accounting for the lack of targeted therapies. Inflammation has been implicated from noninvasive imaging, because of the intense myocardial edema observed on cardiac magnetic resonance imaging 3, 5, and inflammatory changes in the heart have been reported from biopsies of patients, albeit these were taken from the right ventricular myocardium (19). Proteomic analysis of apical cardiac tissue in an experimental model also proposes inflammation might be involved (12), and an increase in macrophage infiltration has been observed 24 h after disease induction (20). To characterize fully the inflammatory aspects of the disease, we used a well-published experimental model of stress-induced takotsubo-like cardiomyopathy, initiated in the absence of ischemic or pathogen-induced inflammatory insults, to follow a detailed time course of inflammatory changes in myocardial tissue, focusing on the infiltration of inflammatory macrophages and the subtypes present, and whether inflammation was localized or systemic. Our experimental model eliminates the complications of any confounding clinical conditions and comorbidities that are found with patients and, for the first time, provides a model whereby we can discriminate and directly compare specific inflammatory responses over a controlled time period. First, we demonstrate a classic inflammatory response upon disease induction, with early infiltration of neutrophils followed by an increase in macrophages and an increase in cytokine gene expression. Second, the increase in myocardial macrophages corresponded to the increase in the percentage of those with a proinflammatory M1 phenotype. Third, the percentage of M2 macrophages did not increase significantly over time, but individual levels correlated with recovery in cardiac function. Fourth, the inflammation appears localized with no substantial increase in circulating cytokine levels; and lastly, the inflammatory cell characterization in our experimental rat myocardial tissue was recapitulated in post-mortem myocardial tissue from takotsubo cardiomyopathy patients.

Macrophage functions help maintain cardiovascular health; however, following an insult, M1 macrophages, which are associated with tissue damage, dominate, and their inflammatory actions can unleash functions that promote disease 13, 14. Subsequently, M2 macrophages produce anti-inflammatory, proangiogenic, and proreparative factors (e.g., vascular endothelial growth factor, and TGF-β1) and engulf apoptotic cells to facilitate repair 13, 14. Following induction of stress-induced takotsubo-like cardiomyopathy, the infiltrating macrophages show a temporal increase in their M1 activation and proinflammatory characteristics peaking at day 4 with a second peak at day 7, a time point where there was a corresponding decrease in the percentage of M2-activated cells. A normal heart has its own tissue-resident cardiac macrophages that display a M2-like, CD163-positive phenotype (21). The drop in percentage of CD163-positive macrophages may reflect exhaustion of these tissue-resident cells after myocardial injury and the replacement by monocyte-derived macrophages infiltrating from the circulation 22, 23. The failure of an up-regulation in the percentage of M2 macrophages at days 5 to 14 strongly contrasts with that observed with models of MI where there is a peak of M1 macrophages and a robust increase in systemic inflammatory cytokine levels. This is followed by increased M2 macrophages (days 5 to 7) that initiates myocardial remodeling and potentially scarring 24, 25. By contrast, in our takotsubo-like experimental model, we observe a peak of M1 macrophages, but no secondary peak of M2 reparative macrophages, and low and no significant increase in circulating cytokine levels. This lack of the timely suppression and spatial containment of the inflammatory response to the myocardium in our takotsubo-like model may perpetuate into a long-term low-grade chronic inflammatory state. This supports our recently demonstrated clinical concept of continued and persistent symptoms rather than complete resolution of disease observed in takotsubo patients 26, 27. Indeed, we show that the increase in percentage EF, as a measure of cardiac function, correlated positively with the percentage of M2 macrophages. This suggests that shifting the balance from M1 to M2 macrophages by specific therapies may be beneficial by promoting myocardial repair and functional improvement post-takotsubo. These ideas need to be validated in future pre-clinical experiments.

In our study, we did not set out to determine whether the inflammatory response observed was the initial cause of the disease or a response to an insult. Given that the greatest drop in cardiac function (as determined by percentage EF) is immediately after disease induction and precedes the peak of neutrophil and macrophages into the myocardium, we consider that the nonresolving inflammation is a secondary feature of disease. It remains unclear what would initiate the inflammatory response in the human disease although mechanical or catecholamine-induced myocyte stress in the akinetic/dyskinetic myocardial regions has been suggested as a potential cause 17, 18, 19. Regardless of the instigating trigger, the persistent presence of proinflammatory cells such as M1-type macrophages would be expected to contribute to the longer-term pathology.

As with rat myocardium, myocardial tissue from Patient #2, deceased 5 days after diagnosis, revealed more prominent infiltrates of macrophages when compared with Patient #1 deceased 5 h after diagnosis, suggesting a primary insult in humans could also result in a progressive inflammatory response, albeit a greater number of post-mortem studies are required to confirm this. Our takotsubo post-mortem hearts exhibit a predominance of M1 proinflammatory macrophages that could initially be dismissed as being associated with other cardiac pathologies, such as coronary artery disease. In the setting of MI, it is well accepted that macrophage infiltrates track to a characteristic localized area of ischemia-induced injury/scarring. By contrast, our findings suggest that in takotsubo, localized M1 macrophage infiltrates occur within viable myocardial areas. This may be a potential marker of takotsubo even when other well-documented cardiac pathologies are present. Imaging of macrophages by ultrasmall superparamagnetic iron oxides in the myocardium of takotsubo patients in the acute phase could provide further indications.

Myocardial biopsies from takotsubo cardiomyopathy patients have previously supported the idea of a potential inflammatory response. In a study by Wittstein et al. (28), of the 5 patients with takotsubo cardiomyopathy who underwent right ventricular endomyocardial biopsy, 4 had interstitial infiltrates consisting primarily of mononuclear lymphocytes and macrophages and contraction bands without myocyte necrosis. A further histopathological analysis of a myocardial biopsy sample obtained from a 71-year-old woman, who died 8 h after onset of takotsubo, revealed multiple necrotic lesions, monocytes and neutrophil infiltration, and hemorrhage (29), whereas the presence of inflammatory cells (mainly monocytes) and disrupted myofibers has also been reported 30, 31. Unlike our current study, however, none of these studies definitively compared biopsies with those of control subjects with no underlying heart pathology, or defined the nature of the inflammatory cells or phenotype of macrophages.

There are no consensus recommendations for long-term management of takotsubo cardiomyopathy to prevent recurrences, and the current clinical management of these patients presents significant clinical equipoise. Long-term treatment with β-blockers, or calcium channel blockers and aspirin did not improve left ventricular function in a multicenter retrospective study (32). From a translational perspective, our results point to an inflammatory component, suggesting that immune-modulation therapies may be a therapeutic avenue in this condition, and intervention studies should be considered.

### Study limitations

The experimental model used is a surrogate and may not fully reflect clinical human takotsubo cardiomyopathy where there are multiple predisposing and/or precipitating factors. Catecholamines have been described as a precipitating factor in human takotsubo development, but they are not the only one. This is currently the only well-published model for this stress-induced disorder, providing us with a unique opportunity to temporally assess the inflammatory response in the absence of multiple predisposing factors and comorbidities that can complicate the analysis in patients. The number of post-mortem cases with Takotsubo cardiomyopathy in this study was limited to 2 patients, and therefore will require confirmation in larger post-mortem datasets.

## Conclusions

We demonstrate for the first time that a classic inflammatory response with predominant M1 macrophage infiltration of the myocardium, without a conventional switch to M2 macrophages is a characteristic of experimental takotsubo-like cardiomyopathy. This results in low-grade chronic inflammation in myocardial tissue that has potential to drive the long-term and persistent symptoms observed in takotsubo patients. Similar inflammatory changes were observed in otherwise macroscopically normal myocardium of post-mortem takotsubo human hearts, pointing to a potential new marker of this condition and suggesting inflammatory modulators, for example, switching the balance from M1 to M2 macrophages may be a therapeutic option.

Perspectives **COMPETENCY IN MEDICAL KNOWLEDGE:** Low-grade myocardial inflammation is a distinguishing feature following both experimental and clinical stress-induced takotsubo cardiomyopathy. Our results establish that a proinflammatory (M1 macrophages) phenotype prevails following a decline in cardiac function, which may contribute toward the morbidity and increased mortality associated with this condition in a subset of patients.

**TRANSLATIONAL OUTLOOK:** No current therapy exists for takotsubo cardiomyopathy. Our study suggests that targeting the myocardial inflammatory process at the onset of disease may have merit as a clinical intervention to alleviate the long-term persistent symptoms that occur in a number of patients.
