# Supplementary material for: Characterization of the Myocardial Inflammatory Response in Acute Stress-Induced (Takotsubo) Cardiomyopathy
Source: JACC Basic Transl Sci. 2018 Dec 31;3(6):766–78. doi: 10.1016/j.jacbts.2018.08.006 (PMC6314973; doi:10.1016/j.jacbts.2018.08.006)
Supplement: Supplemental Figures 1–4 [file mmc1.pdf]

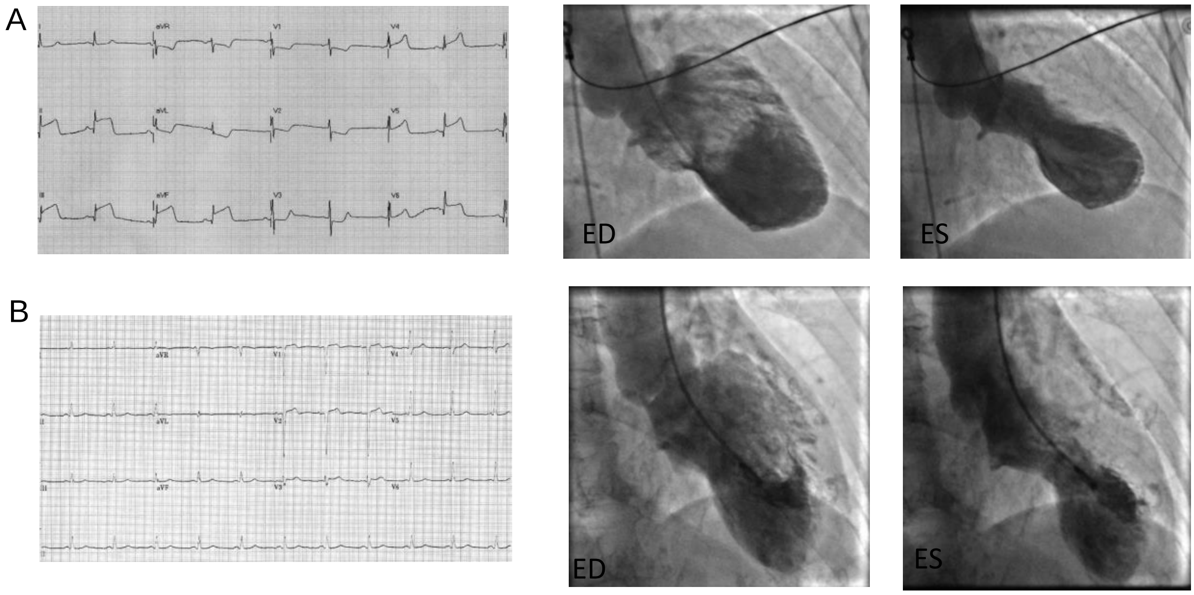

Online Supplementary Figure 1 ECG (A) from patient 1 deceased 5 hours after presenting with chest pain shows 9 mm ST-elevation in inferior and ST-depression in the corresponding precordial leads. End-diastolic (ED), end-systolic (ES) frames of left ventricular angiogram showing apical ballooning. ECG (B) from patient 2, deceased 5 days after presentation shows anterior ST elevation; ED and ES frames of LV angiogram showing apical ballooning.

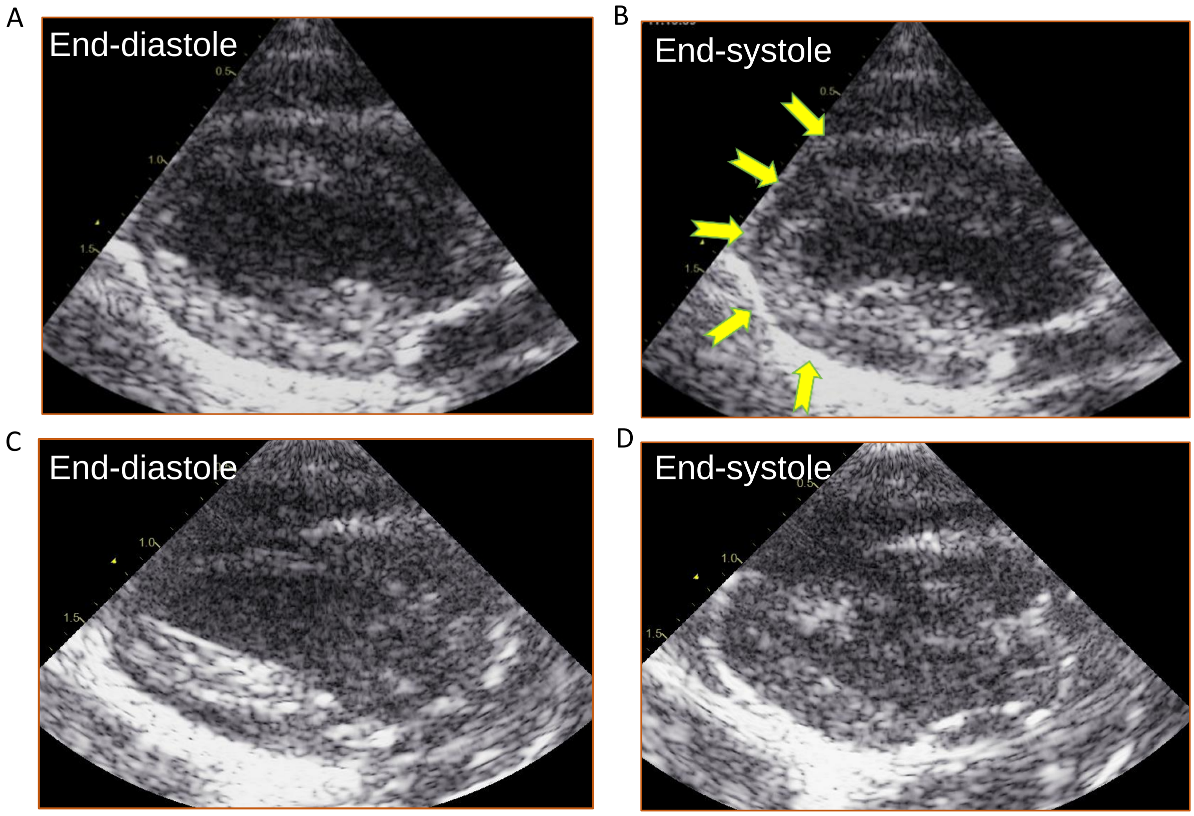

Online Supplementary Figure 2 Characteristics of experimental stress induced cardiomyopathy. Animals were echoed at baseline (before isoprenaline/saline injection) and immediately prior to sacrifice, at day 0-14 post-injection. For all animals, apical ballooning was confirmed by visually observing the wall motion abnormality in each case (consensus agreement between two experienced operators, JS and DD) and confirmed by calculating ejection fractions. Representative examples of end-diastolic and end-systolic frames of a Tako-tsubo rat (day 2) showing apical ballooning (indicated by yellow arrows) (A, B) and a control rat, (C, D) after isoprenaline/saline injection are shown The calculated average pre and post injection left ventricular ejection fractions for Tako-tsubo and control rats sacrificed days 0 through to 14, respectively, were: 79% to 61% (day 0), 79% to 63% (day 1), 72% to 67% (day 2), 72% to 68% (day 3), 74% to 65% (day 4), 74% to 65% (day 5), 78% to 61% (day 6), 74% to 65% (day 7) and 75% to 67% (day 14) compared to 72% to 69% on average for control animals (interaction between Tako-tsubo vs controls and pre to post injection with a p=0.04 showing a pre to post change that is significantly different in Tako-tsubo animals vs controls).

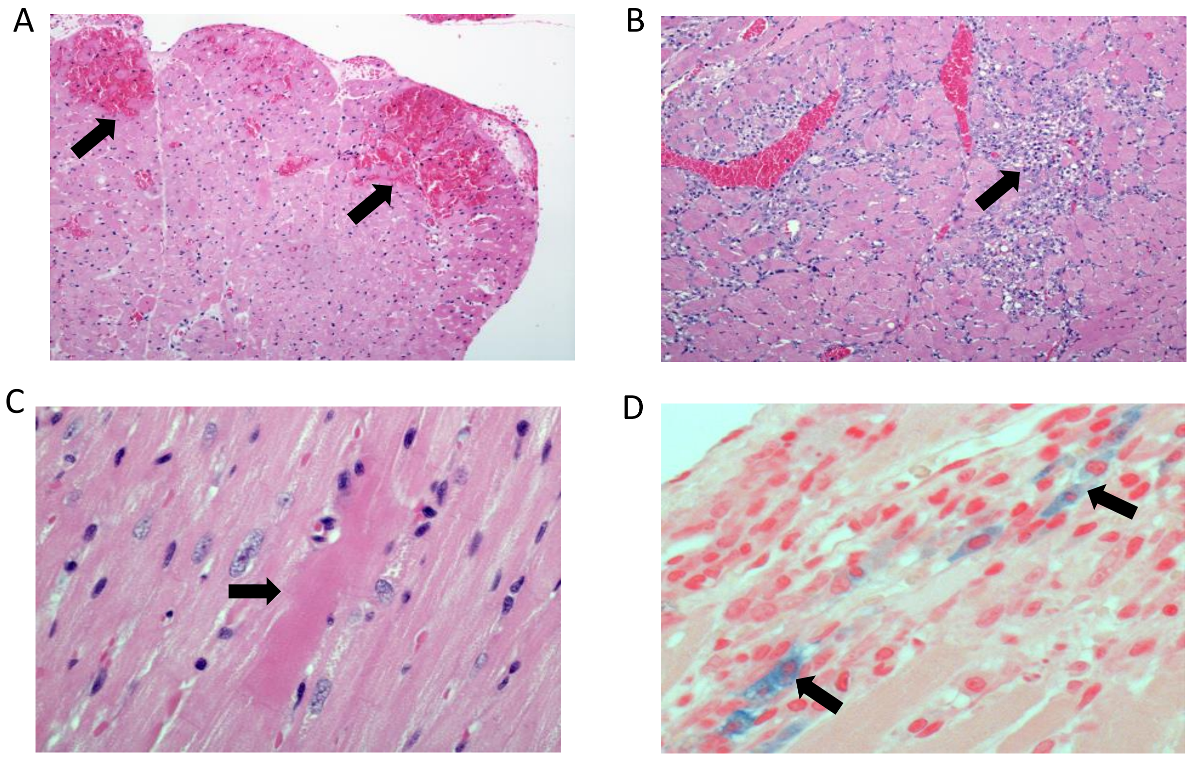

Online Supplementary Figure 3. Histological characterization of heart tissue was defined by a scoring system for hemorrhage, inflammation, degenerate myofibrils and eosinophilic fibers as described in Methods. Representative examples, denoted by arrows as hemorrhage (A); inflammation (B); degenerate myofibrils and eosinophilic fibers (C). Prussian blue stained sections demonstrating hemosiderin and clearance of hemorrhage (D).

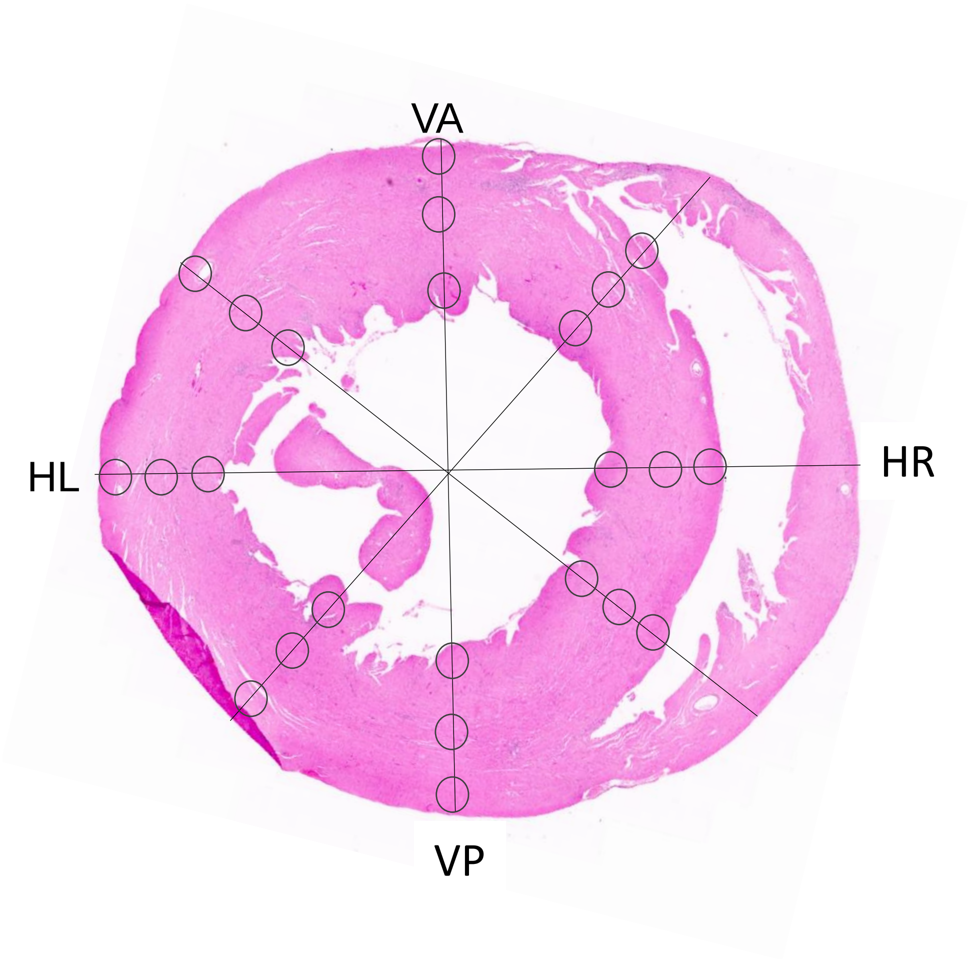

Online Supplementary Figure 4. Schematic representation of areas analyzed for cell counts in heart sections using defined fields of view denoted by circles. Sections were partitioned into 8 consecutive parts. Each section was separated into 3 fields of view (epicardium and mid points and endocardium) to directly compare areas of the heart. An average of cell counts per field of view was recorded. VA, vertical anterior; VP vertical posterior; HL, horizontal left; HR horizontal right.
